# Data for Politics: Creating an International Research Infrastructure Measuring Democracy

**DOI:** 10.1016/j.patter.2020.100056

**Published:** 2020-07-02

**Authors:** Staffan I. Lindberg, Juraj Medzihorsky

**Affiliations:** 1 V-Dem Institute, Department of Political Science, University of Gothenburg, Gothenburg 40530, Sweden

**Keywords:** DSML 5: **Mainstream**: Data science output is well understood and (nearly) universally adopted

## Abstract

Questions such as how democratic a country is, how free are its media, or how independent is its judiciary are highly important to researchers and decision makers. We describe a research infrastructure that produces the world's largest dataset on democracy, governance, human rights, and related topics. The dataset is far more resolved and accurate than previous efforts, currently covers 202 political units from 1789 until the present, and is regularly updated each spring. The infrastructure involves an online survey of over 3,000 experts from 180 countries. Survey design and advanced statistical techniques are crucial for assuring data validity. The infrastructure also provides reports and analyses based on the data and easy-to-use tools for exploring and graphing the data.

## Highlights

• A large research infrastructure with over 3,000 participants from 180 countries
• World's largest democracy dataset, on 202 political units from 1789 until the present
• Regular updates and reports, easy-to-use online tools
• Expert surveys combined with advanced statistical modeling

## The Bigger Picture

V-Dem is a new research infrastructure providing almost 30 million data on democracy, human rights, governance, judiciary, media, and related areas. The data are generated through collaboration involving over 3,000 experts from 180 countries and advanced statistical techniques. The V-Dem democracy indices are far more resolved and accurate than previous efforts.The research infrastructure is built to deliver annual updates of data in coming years and to produce a democracy report in March every year, assessing the trends for democracy and autocracy in the world. The societal impact is already evident, with a wide variety of organizations using V-Dem’s data for benchmarking, strategic decision making, and analysis, such as the World Bank, the European Commission, Open Government Partnership, Mo Ibrahim Foundation, USAID, International IDEA, and Transparency International.

## Teaser

V-Dem is a research infrastructure that produces the world's largest dataset on democracy, human rights, governance, judiciary, media, and related areas. The dataset covers over 200 political units from 1789 until the present. It provides indices far more resolved and accurate than previous efforts, thanks to a collaboration of over 3,000 experts from 180 countries and advanced statistical techniques.

## Introduction

Varieties of Democracy (V-Dem, https://v-dem.net)^1^ provides the world of scholars, students, journalists, policy makers, and practitioners with almost 30 million data on democracy, human rights, political parties, civil society, media, judicial systems, political equality and exclusion, covering some 450 indicators for 202 political units (“countries”) from 1789 to 2019. The data are regularly updated with information on the last year and improved information on the previous years. Since its first public release in January 2016, it has quickly become a standard resource for academic researchers. The data are published fully open-access under a CC-BY-SA license and can be downloaded along with complete documentation from the website. The website also provides 13 online analysis/visualization tools, including a predictive assessment of which countries are at highest risk of becoming autocratic in the coming 2 years.^2^

Why did we start this somewhat unusual undertaking? In the first years of the 21st century, many of us were frustrated by the conceptual narrowness and empirical limitations of the existing democracy measures. The limitations of Freedom House,^3^ Economist Intelligence Unit's Democracy Index,^4^ Polity,^5^ and other measures of democracy were often discussed among social scientists and practitioners. As the number of democracy measures proliferated, several scholars stepped forward to critique them.^6^, ^7^, ^8^, ^9^, ^10^, ^11^, ^12^, ^13^, ^14^, ^15^, ^16^, ^17^ The limitations included questionable and very narrow conceptualizations of democracy; uncertain reliability of sources, ratings, and political considerations; opaque or untested modes of aggregation of source data into indices; and lack of estimates of measurement error, among other things. There were some efforts to produce better indices.^18^, ^19^, ^20^, ^21^, ^22^, ^23^, ^24^, ^25^, ^26^, ^27^, ^28^, ^29^, ^30^, ^31^, ^32^ However, none had all the features desired by scholars and practitioners.^6^, ^7^, ^8^, ^9^, ^10^, ^11^, ^12^, ^13^, ^14^, ^15^, ^16^, ^17^ V-Dem was launched in this context, to provide new data with these features and free of the limitations of the existing measures of democracy.

## Results

The first impulse behind the V-Dem project dates to 2003 and the effort to build the required infrastructure started in earnest in 2008. We can hardly estimate how many work-days or years have gone into it since. V-Dem is now the result of probably the largest-ever social science collaboration with academic colleagues from 180 countries serving as our 5 co-principal investigators; 19 project managers; 26 postdocs, research associates, and PhD students; 28 regional managers; some 150 country coordinators; and over 3,200 country experts. It is a massive, collective effort, including an annual update of the dataset released in early spring every year. In March 2020, version 10 was released.

In the beginning, we expected that the project could be accomplished over 2 to 3 years with a few research assistants, Excel spreadsheets, and less than $1 million. This was a reflection of the standard practices in political science at the time. It proved to be a miscalculation. We ended up having to create a complex research infrastructure consisting of custom-designed web interfaces for data collection, a PostgreSQL database for data handling, a FileMakerPro database for managing questionnaires and respondents, a website for both internal use and social media, a custom-designed Bayesian item response theory measurement model implemented in Stan^33^ custom-designed data quality control and cleaning protocols (many of which use R^34^), and a V-Dem Institute with purposely trained program and data managers with assistants.^35^^,^^36^ We spent over $4.5 million between 2010 and 2016 to make it happen, not counting the thousands of working hours invested by Principal Investigators, Project Managers, and associated researchers supported by their own universities.^37^ The continued operation costs about $1 million annually.

**Figure 1:**
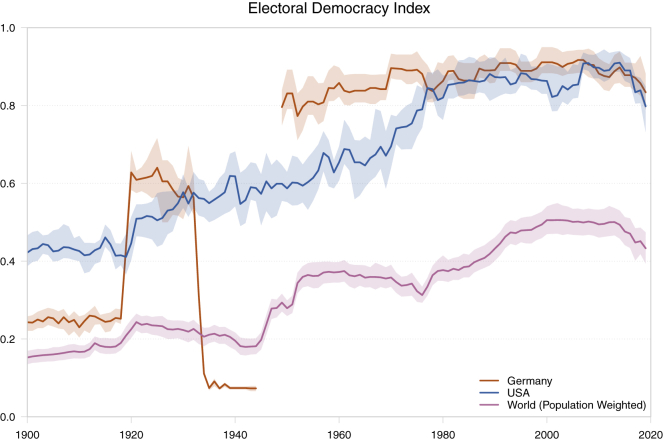
Electoral Democracy in the United States, Germany, and the World 1900 Shaded areas represent 95% intervals. The values for Germany exclude East Germany.

V-Dem is unique in several ways. First, it provides a picture of democracy that is both broader and in higher resolution than other datasets, spanning from 1789 to the present, and including most countries in the world. Second, it is the only data source on democracy that captures the multiple dimensions and conceptions of democracy. Democracy, understood very generally, means *rule by the people.* Beyond this basic feature, there is little agreement. Most democracy measures reflect a very narrow conception. For example, in Polity IV, the United States is rated as fully democratic for most of the 19th century and all of the 20th, disregarding issues, such as slavery, women's exclusion, and so on.^38^^,^^39^ Notably and different from any other democracy measures, according to the V-Dem's electoral democracy index the US does not achieve status as a higher-end democracy until around 1970 after the Civil Rights Movement. This makes it clear that the United States is a relatively young democracy rather than the oldest in the world (Figure 1).

Third, there are competing conceptions of democracy in the literature.^40^, ^41^, ^42^ But indices tend to focus on the electoral aspects and possibly some liberal ones, and have little to say about participatory, egalitarian, or deliberative aspects. The core V-Dem index measures electoral democracy as “polyarchy,” the seminal concept defined by Robert Dahl,^43^, ^44^, ^45^ and its 7 core constitutive components. The other 4 indices measure liberal, deliberative, participatory, and egalitarian democracy (Table 1). Fourth, by providing some 450 indicators and over 50 other indices that aggregate them, V-Dem is the source with the highest resolution on a range of issues associated with democracy and the resulting almost 30 million data are the largest by multiple orders of magnitude.

**Table 1:** Varieties of Democracy

Electoral *Core values:* Contestation, competition *Question:* Are important government offices filled by free and fair multiparty elections before a broad electorate? *Institutions:* Elections, political parties, competitiveness, suffrage, turnover	Deliberative *Core values:* Reasoned debate and rational arguments *Question:* Are political decisions the product of public deliberation based on reasoned and rational justification? *Institutions:* Media, hearings, panels, other deliberative and consultative bodies
Liberal *Core values*: Individual liberty, protection against tyranny of majority and state repression *Question*: Is power constrained and are individual rights guaranteed? *Institutions*: Civil liberties, independent bodies (media, interest groups); separation of powers, constitutional constraints on the executive, strong judiciary with political role	Egalitarian *Core values:* Equal political empowerment *Question:* Are all citizens equally empowered to use their political rights? *Institutions:* Formal and informal practices that safeguard or promote equal distribution of resources and equal treatment
Participatory *Core values:* Direct, active participation in decision making by the people *Question:* Do citizens participate in political decision making? *Institutions:* Voting, civil society, strong local government, direct democracy instruments	

The fifth distinctive feature is captured by the V-Dem motto, “Global Standards, Local Knowledge.” We rely on more than 3,200 academics and other experts to rate countries. More than 64% of these experts are nationals of or residents in the primary country they rate. In a real sense, each country's experts rate their own country. At the same time, our broad concepts and complex methods to blend in-country and cross-national ratings help ensure international comparability. We do not consider these experts' ratings “subjective.” We have worked long and hard to validate each person's expertise on a particular domain in the country or countries they rate, as well as their independence and sincerity. The V-Dem approach then asks them to answer very specific questions on the existence of rights and institutions de facto on the ground. Each question is answered by multiple raters. In aggregating the ratings, we pay particular attention to assuring comparability across countries and over time. The main challenge comes from that even the most carefully designed scale may not be used in the same way by every rater. We overcome it by combining survey design with statistical modeling. Our surveys incorporate vignette questions that help us to anchor the answers to a common scale, and we aggregate the responses with a custom-designed Bayesian item response model.^46^^,^^47^ The model not only aggregates the ratings into indices, but also numerically summarizes the uncertainty attached to them.

V-Dem provides directly observable indicators too, such as what is in the constitution or whether an election was held in a given year. Those indicators make about half of the V-Dem data. Yet the other half of evaluative indicators, all based on country expert assessments, are critical and arguably more valuable. They are seeking to measure 2 kinds of phenomena. First, there are those that cannot be meaningfully evaluated by inspecting provisions without considering their actual implementation. Take, for example, the power of legislatures to hold the executive accountable, which is a critical aspect of any democratic government. Most countries in the world now have elected legislatures that have this power by constitutional or other provisions—even North Korea. By that objective measure a host of authoritarian governments are as democratic as is say Sweden, France, or the United Kingdom. What matters is if and to what extent the legislature actually uses this power in practice. This is something country experts with intimate knowledge of legislatures have relatively accurate and reliable knowledge about.

**Figure 2:**
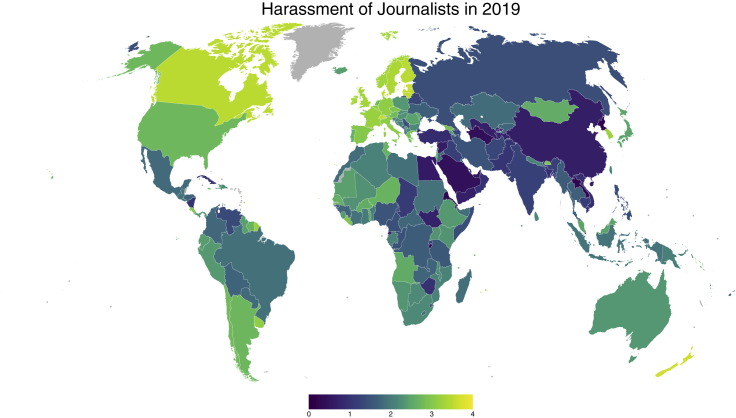
Journalists' Freedom from Harassment in 2019 Higher values mean more freedom.

**Figure 3:**
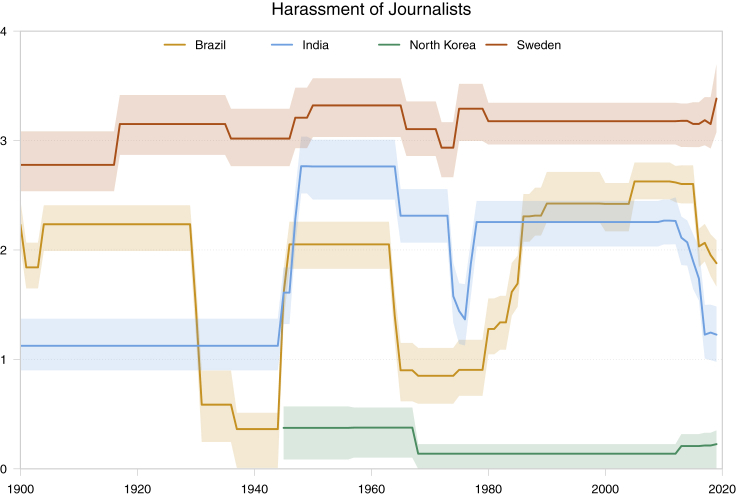
Harassment of Journalists in 4 Countries, 1900–2018 Lower values indicate more harassment. Shaded areas indicate 95% intervals.

The second kind may be even more intriguing and important. For example, media freedom requires many things, including that journalists can report critically about the government without being harassed and intimidated into silence (Figure 2). We could, say, count the number of journalists harassed and perhaps even killed every year in each country to get a hard, objective measure. The problem is that the number would be zero (most of the time) for a country like Sweden, but also for one like North Korea. We are faced with empirical equivalence of 2 substantively opposite situations. An increase in the number of journalists being harassed in Sweden would probably mean that things are getting worse. Yet, an increase in North Korea would in all likelihood mean that the regime is liberalizing so that journalists actually dare writing something critical about the government in the first place. What we really want to know is an unobservable that, yet again, country experts can assess with high reliability: *if* a journalist reports something critical of the government, how likely is it that s/he will be harassed or worse? Such measures are V-Dem's most unique feature. Figure 3 shows the V-Dem data for this indicator and 4 countries: Sweden where it fluctuates a little at the very high end of the scale; North Korea at the very bottom; and Brazil and India where variation is great over time and accurately captures the very worrying ongoing “autocratization” under president Bolsonaro and prime minister Modhi, respectively.

## Discussion

Since its first public release in 2016, the V-Dem data have become a standard go-to for researchers working on a variety of topics. As of time of this writing, it has 550 citations on Google Scholar, of which 224 are from 2019. Not all of them are in the domain of political science. To give just a few examples, the data have been recently used in research on population health,^48^ demography,^49^ communication,^50^ migration,^51^ environmental conservation,^52^ or cultural evolution.^53^ This is thanks to the fact that it contains numerous indices that go beyond the narrow issues of political regimes. The indices cover both broad issues, such as educational and health equality or academic freedom, and more specific ones, such as media corruption or antisystem movements. Furthermore, V-Dem aids the Digital Society Project^54^ by collecting and curating data using its infrastructure. This data provide indices of issues, such as online censorship, fake news dissemination, or social media polarization, and is also available integrated with the V-Dem data. Regardless of the domain, researchers who use V-Dem data are often interested in a relationship between 2 factors, and either one of them, or some other factor that modifies or confounds the relationship, is measured by V-Dem. Another common use of the V-Dem data is in explaining some outcome, and either the outcome and/or the candidate causes are measured by V-Dem. The V-Dem data can be also applied in forecasting, by providing features that improve forecast accuracy, or in-depth country or regional studies where it provides background information in a comparative format. In short, if a research question involves in some way the political regime or a broad spectrum of related topics, there is a good chance V-Dem data may help in answering it.

Furthermore, the V-Dem data are used by a set of actors outside of academia, and we are regularly invited to support and advise in these efforts. To mention just a few examples the World Bank used data for the *World Development Report 2017* and continues to use V-Dem data extensively, including as a data source for the World Governance Indicators; USAID have replaced Freedom House data with V-Dem indicators to benchmark which countries are eligible for budget support from the United States; Article 19 use our data on media for their annual report on global expression; UNDP's report *The Indicators We Want* included some 60 V-Dem indicators to be supplementary measures of SDG 16 targets for countries in the world; Transparency International use our corruption data as source in their annual assessment; International IDEA's *Global State of Democracy* builds to 70% on V-Dem data; the Mo Ibrahim Foundation use a series of our indicators in their Index of African Governance; and the European Commission/DEVCO require all missions to use V-Dem data for their annual risk assessment framework. At the V-Dem Institute, we also produce our own annual democracy report on the state and trends for democracy and autocracy in the world, which is used extensively as an authoritative account in the policy-practitioners’ community.

We hope you will find the V-Dem infrastructure and the resulting 30 million data on democracy interesting and worthwhile engaging with. The V-Dem Institute will continue to collect data every year and therefore also to go on re-engaging the network of country experts. The hope is to be able to publish an update dataset every March for many years to come.

## Experimental Procedures

### Resource Availability

#### Lead Contact

Further information and requests for resources should be directed to and will be fulfilled by the Lead Contact, Juraj Medzihorsky (juraj.medzihorsky@v-dem.net).

#### Materials Availability

This study did not generate new unique reagents.

#### Data and Code Availability

The current version of the V-Dem Dataset (V10) is available at https://doi.org/10.23696/vdemds20.

The protocols used in data collection and processing are described in documents available at https://www.v-dem.net/en/data/reference-materials-v10/.
